# Fast Water Desalination with a Graphene–MoS_2_ Nanoporous Heterostructure

**DOI:** 10.1021/acsami.4c01960

**Published:** 2024-05-20

**Authors:** Omid Barati Farimani, Zhonglin Cao, Amir Barati Farimani

**Affiliations:** † Department of Mechanical Engineering, Carnegie Mellon University, Pittsburgh, Pennsylvania, 15213 United States; ‡ Department of Chemical Engineering, Carnegie Mellon University, Pittsburgh, Pennsylvania 15213, United States

**Keywords:** water desalination, heterostructure membrane, bilayer membranes, improved water flux, high ion injection rate

## Abstract

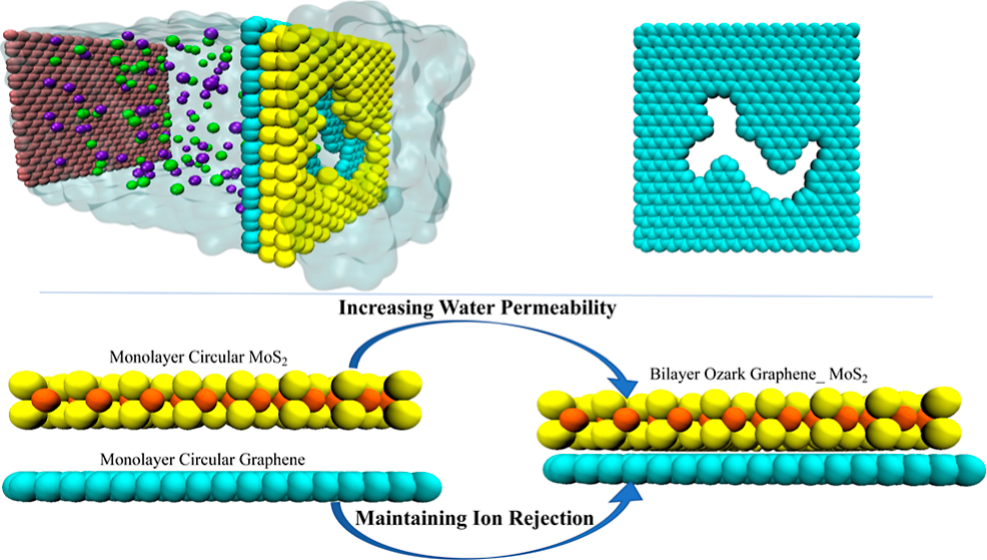

Energy-efficient water desalination is the key to tackle the challenges with drought and water scarcity that affect 1.2 billion people. The material and type of membrane in reverse osmosis water desalination are the key factors in their efficiency. In this work, we explored the potential of a graphene–MoS_2_ heterostructure membrane for water desalination, focusing on bilayer membranes and their advantages over monolayer counterparts. Through extensive molecular dynamics simulation and statistical analysis, the bilayer MoS_2_–graphene was investigated and compared to the monolayer of graphene and MoS_2_. By optimizing the heterostructure membrane, improved water flux was achieved while maintaining a high ion rejection rate. Furthermore, the study delves into the physical mechanisms underlying the superior performance of heterostructure nanopores, comparing them with circular bilayer and monolayer pores. Factors investigated include water structure, hydration shell near the membrane surface, water density, energy barrier using the potential of mean force, and porosity within the nanopore. Our findings contribute to the understanding of heterostructure membranes and their potential in enhancing the water desalination efficiency, providing valuable insights for future membrane design and optimization.

## Introduction

In recent years, severe droughts have ravaged all the world, leaving behind significant socioeconomic, environmental, and ecological consequences.^1−5^ Providing fresh water for the world population has become more challenging due to global warming and deprecation of water resources. Water desalination through reverse osmosis (RO) can be a viable solution to provide fresh water to people since 71% of the earth’s surface is covered with saline seawater.^6−8^ However, this method still has the drawback of high energy consumption due to the low water permeation rates of traditional polymeric or zeolite membranes.^8,9^ The ultrathin nature of 2D materials facilitates the accelerated permeation of water molecules through nanopores, resulting in a more energy-efficient RO water desalination process.^10−15^ Nanoporous graphene (NPG) membranes exhibit water flux rates that are 2–3 orders of magnitude higher than conventional RO membranes, while maintaining a high percentage of salt rejections.^16−19^ Furthermore, the exceptional mechanical strength exhibited by 2D materials enables them to withstand the pressures encountered during RO desalination operations.^14,20^ Recently, some studies presented a comprehensive analysis of how the presence of multiple layers, the distance between layers, and the alignment of pores affect the desalination performance of NPG membranes.^21−24^ The ion rejection rate and water permeation in nanopores used for water desalination can also be influenced by geometrical and material factors of nanopores.^25,26^ The shape and size of the pores play a critical role in determining the efficiency and effectiveness of the desalination process;^27−35^ however, the challenge in optimizing the membrane is the trade-off between ion rejection and permeation rate. Maximizing the permeation rate sacrifices the ion rejection efficiency. Single-layer graphene has shown superior permeation with an optimized ozark pore^35^ and single-layer MoS_2_ with hydrophilic Mo edges has superior permeation compared to other single-layer MoS_2_ with sulfur (S) at the pore edges.^10^ The question we are asking is “How can we take advantage of both MoS_2_ and graphene permeation enhancement properties simultaneously?”

To respond to this question and in the quest for efficient water desalination, we created graphene–MoS_2_ heterostructure membranes and investigated their desalination efficiency. We explored different configurations of the heterostructure and investigated the underlying physical mechanisms contributing to its superiority. By analyzing interfacial water density, energy barriers for water and ion transport, and water and ion distribution [kernel density estimate (KDE)] within the nanopores, we uncover the key factors driving its remarkable performance. Additionally, we quantitatively demonstrate the impact of nanopore geometry on water desalination by highlighting the ion rejection achieved by smaller hydraulic diameters and a large porosity. To achieve energy-efficient water desalination, it is crucial for the nanopore to strike a balance between two key factors: facilitating a high water flux and maintaining a high rate of ion rejection. The ideal nanopore design should enable the rapid passage of water molecules through the pore, ensuring that a high volume of water can be processed efficiently. At the same time, the nanopore should effectively block or remove ions, preventing their passage and maintaining a high level of desalination performance. Thus, it is important to select the atoms around the pore (engineering pore) in order to increase the water flux while maintaining the ion rejection. By benchmarking and studying the performance of heterostructure membranes and comparing with circular structures (which provide higher water flux and the lowest salt rejection among triangular, rhombic, and rectangular cases^31,35^) with varying geometries and material combinations, valuable insights can be gained to guide the development of more efficient water treatment technologies.

## Methods

**Figure 1:**
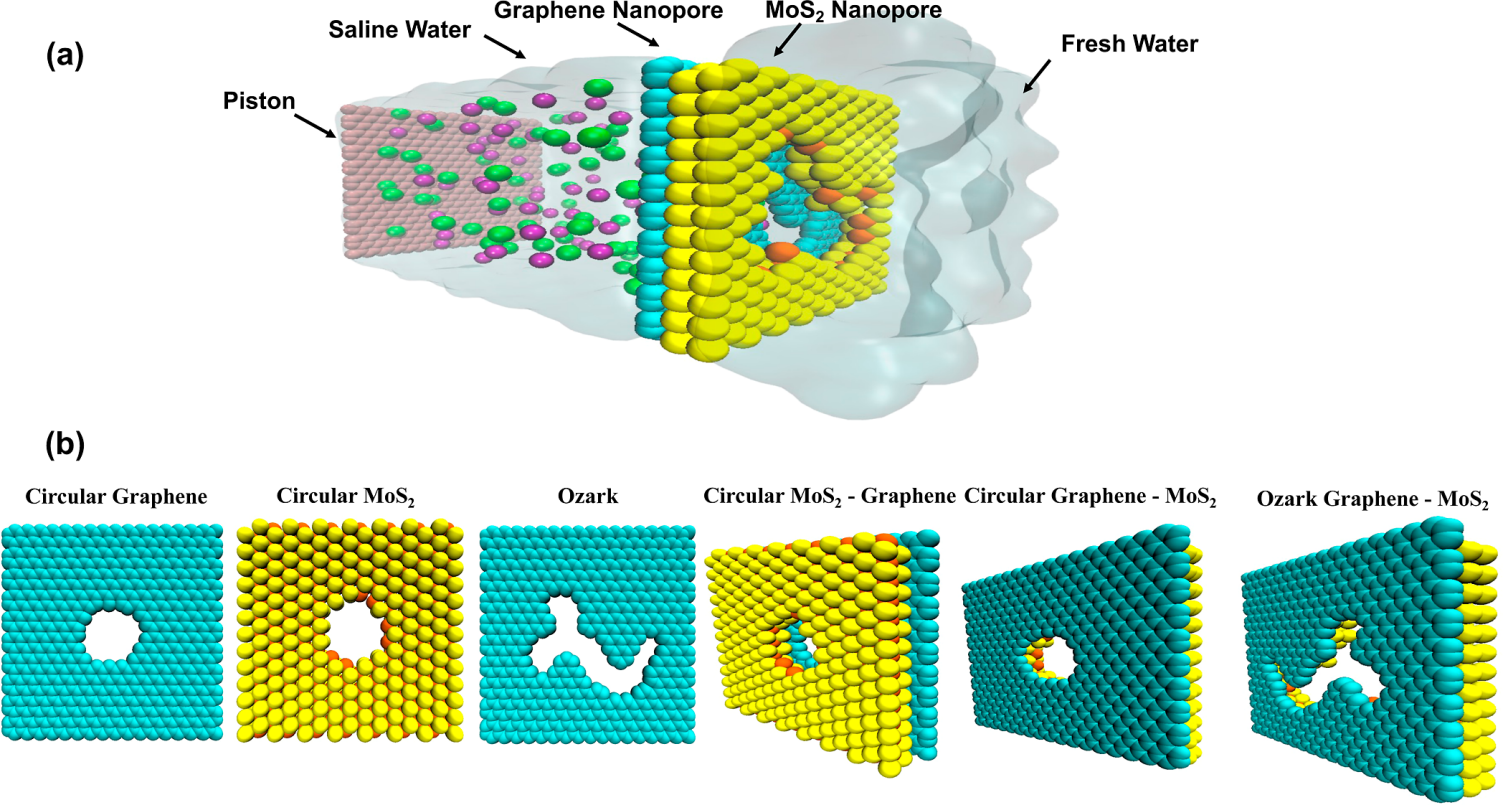
(a) Water desalination system featuring a heterogeneous nanoporous membrane simulated using molecular dynamics (MD). In this system, a piston is used to apply pressure on saline water, pushing it toward the membrane. The nanopore effectively filtered out ions, enabling water molecules to permeate through and reach the fresh water side. (b) Series of snapshots capturing the simulated circular and ozark shapes in graphene and MoS_2_ nanopore membranes investigated in this study.

The simulation system consisted of a graphene piston, saline water section, NPG and MoS_2_ membrane, and pure water section (Figure 1a). The simulation environment is a periodic box with dimensions approximately 4 nm × 4 nm × 13 nm in the *x*, *y*, and *z* directions. To create a heterogeneous membrane, we added a layer of MoS_2_ to the graphene layer. We studied different nanopore geometries including circular graphene, circular MoS_2_, ozark, circular MoS_2_–graphene, circular graphene–MoS_2_, and ozark graphene–MoS_2_ (OGM) and ozark graphene–MoS_2_ engineered (OGME) nanopore area shapes (Figure 1b). OGM and OGME differ in 3 atoms. OGME has slightly larger nanopores (3 more atoms were removed from OGM to make OGME). The 3 atoms were removed at the designated area depicted in the Supporting Information (Figure S2). In order to create these nanoporous membrane systems, we used visual molecular dynamics (VMD)^36^ and atomic simulation environment (ASE)^37^ software. The graphene and MoS_2_ membranes were placed between the saline and fresh water sections. A graphene piston was placed behind the saline water section. The piston is used to apply external pressure to the saline water. The monolayer NPG (MNPG) and bilayer NPG (BNPG) membranes allowed water and ions to pass through the pores into the filtered water section. In order to separate the rigid graphene and MoS_2_ layers from each other, the interlayer space between S (sulfur) and C (carbon) is defined to be 3.51 Å. We adjust the distance based on the experimental observation of the distance between MoS_2_ and graphene.^38^ The cyan color represents the graphene (carbon atoms) membrane, and the MoS_2_ is represented by yellow (S atoms) and orange (Mo atoms) colors (Figure 1b).

The simulation comprises a variable number of atoms (∼14,700 atoms, depending on the geometry of the graphene and MoS_2_ nanopore), and the saline water contains potassium chloride (KCl) with an estimated molarity of approximately 2.28 M. In order to see the effect of the salt type on the desalination performance, we performed 12 simulations (for 3 pressures, each for 4 seeds) for OGM. We observed a small drop in the flux of water in the NaCl case, while the ion rejection is almost similar for KCl and NaCl. Ion rejection is a bit smaller at lower pressures due to the smaller size of Na^+^ ions (see Figure S5a,b in Supporting Information). Based on our previous studies^13,14^ on the effect of salt concentration in water desalination, the salt concentration has an insignificant effect on both ion rejection and water flux. The desalination performance of MNPG and BNPG with various system parameters was predicted through MD simulations using the LAMMPS package.^39^ The system initially underwent energy minimization and equilibration stages. The temperature was set to 300 K, and water molecules were assigned random Gaussian velocities. Subsequently, an *NVT* ensemble was simulated for 10 ns as the production stage, during which the trajectories of the molecules were recorded for analysis.

To maintain a constant temperature of 300 K, a Nosé–Hoover^40,41^ thermostat with a time constant of 0.5 ps was employed. The system is then simulated for 5 ps until equilibrium is reached. The SPC/E^42^ water model with constrained bonds and angles using the SHAKE algorithm was used to model water molecules (set to a 0.01% accuracy tolerance). Lennard-Jones (LJ) potentials with long-range Coulombic interactions were applied for atomic interactions, and LJ potentials were calculated using the arithmetic mix rule for interactions between different elements^10,43,44^ (see Supporting Information Table S1) and the periodic boundary conditions were applied to all directions. The particle–particle particle-mesh^45^ solver with a 0.001% root-mean-square (rms) error was used for computing long-range Coulombic interactions. The cutoff radius for both LJ and Coulombic interactions was set to 12 Å. In each simulation, the initial step involves minimizing the system energy through 1000 iterations using the steepest descent algorithm.^46^ The water molecules’ initial velocity is determined by generating random variables with a Gaussian distribution at a simulation temperature of 300 K. In the simulation, both the piston and the nanopore membranes are treated as rigid bodies, and the interactions between atoms within them are not computed for computational efficiency reasons. To achieve a state of equilibrium, the *NPT* (isothermal–isobaric) ensemble is applied to the entire system with the temperature set to 300 K and the pressure set to 1 atm. To induce the necessary pressure drop, an external pressure was exerted on the water molecules along the *z* direction. The pressure drop was achieved by varying the applied pressure value, which ranged from 100 to 200 MPa (Figure 1a). Since the ion permeation events significantly reduce at lower pressures (6–10 MPa) in MD simulations and require very long simulations, we applied larger pressures to observe more ion permeation events in a smaller simulation time horizon. In previous reports, it has been shown that the applied pressure does not affect the membrane performance.^11,14,35^ The force (*F*) applied can be determined by multiplying the applied pressure (Δ*P*) by the area (*A*) of the piston. Additionally, the number of water molecules (*n*) involved in the system is a factor in this equation.

To calculate the areas of graphene nanopores, a computer vision method utilizing the Open CV package^47^ is employed (see Supporting Information Table S2). A visualization plot is generated for each NPG membrane, utilizing all atoms within the 0–40 Å range in both the *x* and *y* dimensions (depends on the atom type and the radius of atoms in membranes; the dimensions of *x* and *y* may slightly vary) (see Supporting Information Figure S1a,b).

## Results and Discussion

**Figure 2:**
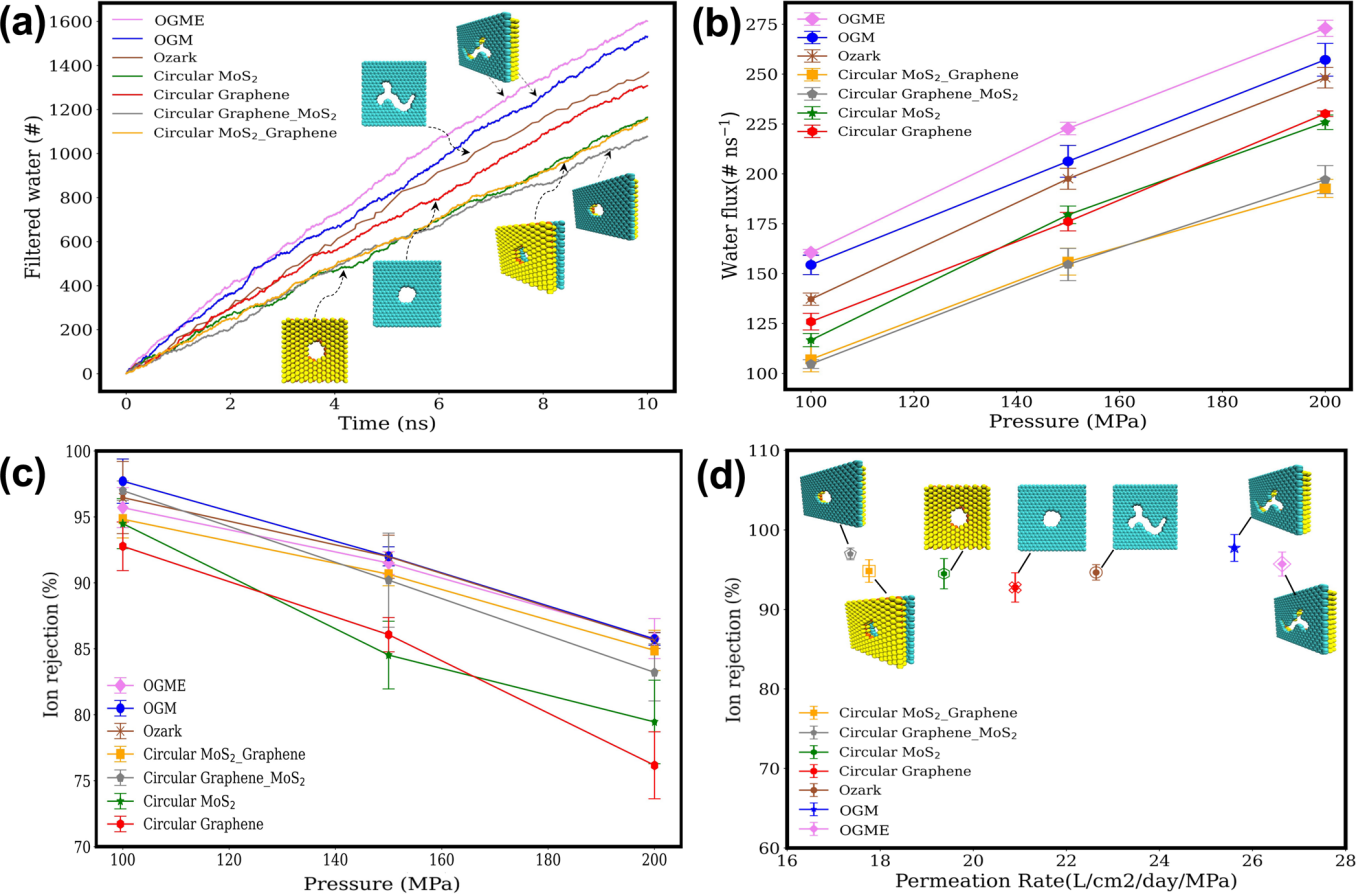
(a) Number of filtered water molecules versus time (10 ns) in seven different nanoporous membranes under an external pressure of 100 MPa. (b) The water flux was studied in relation to the external water pressure ranging from 100 to 200 MPa. (c) Ion rejection with respect to external water pressure for seven various nanopore membranes. (d) The relationship between permeation rate and ion rejection percentage was examined for different nanopores, specifically focusing on an external pressure of 100 MPa to calculate the ion rejection.

The nanopore’s water desalination performance is typically assessed using two key metrics: water flux and ion rejection rate. These metrics serve as fundamental measures to evaluate the effectiveness of a nanopore in separating water and ions during the desalination process. The water flux quantifies the volume of water passing through the nanopore per unit time, while the ion rejection rate represents the percentage of ions that are successfully blocked or removed by the nanopore. First, the MD simulation trajectories were postprocessed to calculate the time-dependent filtered water molecules (Figure 2a, for 100 MPa). By calculating the slope of the curves for filtered water per unit time, the water flux can be obtained for each pressure.

Under an external pressure of 100 MPa, the water flux values for different nanopore configurations are as follows: OGME (160.56 molecules/ns), OGM (154.4 molecules/ns), ozark (136.46 molecules/ns), circular graphene–MoS_2_ (104.66 molecules/ns), circular MoS_2_–graphene (107.05 molecules/ns), circular MoS_2_ (116.67 molecules/ns), and circular graphene (125.93 molecules/ns). The water flux generally increases linearly with an applied external pressure. The higher water flux observed in the OGM nanopore suggests its potential for maintaining a superior performance even at lower pressures in real-world RO water desalination plants (as depicted in Figure 2b). At higher pressures like 200 MPa, water flux maintains a superior performance (272.92 molecules/ns) for OGM among other nanopores.

The membrane’s salt rejection capability plays a crucial role in its overall performance. The percentage of ions rejected by different pore sizes and layers in the heterostructure and circular pore areas of the membrane are depicted in Figure 2c. Each data point represents the average value for 4 simulations conducted and averaged at the same pressure, with error bars indicating one standard deviation based on four simulations conducted for each nanopore. Notably, the OGM nanopore exhibits an excellent average ion rejection of 97.71% under a 100 MPa pressure while the ion rejection for ozark^35^ with the same pore area is lower (94.76%). The OGME nanopore with a larger pore area size exhibits a slightly lower ion rejection rate compared to the OGM nanopore. This highlights how the narrow interlayer region in bilayer graphene–MoS_2_ serves as an energy barrier for ions. Comparative simulations of circular graphene–MoS_2_ and circular MoS_2_–graphene membranes demonstrate that circular graphene–MoS_2_ exhibits higher ion rejection (96.99% compared to 94.83%, respectively) while maintaining a generally consistent water flux. Furthermore, simulations of monolayer MoS_2_ and monolayer graphene reveal that MoS_2_ outperforms graphene in terms of ion rejection (94.49% compared to 92.77%), although it falls slightly behind bilayer circular graphene–MoS_2_ with the same pore area.

Next, we computed the permeation rates of different membranes. A higher permeation rate indicates a faster flow of water through the membrane, which is desirable for efficient water desalination systems. Permeation rate is influenced by factors such as membrane properties, applied pressure, pore size, and membrane surface area.^48^

The permeation rate for OGME, OGM, ozark,^35^ circular MoS_2_–graphene, circular graphene–MoS_2_, circular MoS_2_, and circular graphene nanopores is 26.64, 25.61, 22.64, 17.76, 17.36, 19.37, and 20.90 L/cm^2^/day/MPa, respectively (Figure 2d). Both bilayer circular graphene–MoS_2_ nanopore and bilayer OGME nanopore have almost the same ion rejection rate; however, the bilayer OGME pore has a 53.5% higher permeation rate compared with the bilayer circular graphene–MoS_2_ nanopore. Moreover, when compared with the monolayer ozark nanopore, the OGM nanopore with the same pore area has a better water permeation rate (13%) and the ion rejection is 3.2% more under a 100 MPa external pressure (Figure 2c,d). It has to be mentioned that the irregular slim shape of the bilayer OGM nanopore outperforms circular monolayer graphene and MoS_2_ nanopores in terms of ion rejection (5.1 and 3.3% higher) and permeation rate (22.5 and 32.2% higher under the same pressure). In addition, from the point of view of selectivity, when we compare the OGME pore area with the OGM, we see a 4% increment in water permeation, while ion rejection decreased by only 2%. It has to be mentioned that when we take the average of water flux rate from all 4 simulations at 100, 150, and 200 MPa, we see that the water flux increases by 5.66% in the OGME pore, while ion rejection remains almost constant (decreases only by 0.87%). It means that the OGME pore has better ion rejection performance at higher pressures (see Supporting Information Figure S2).

**Figure 3:**
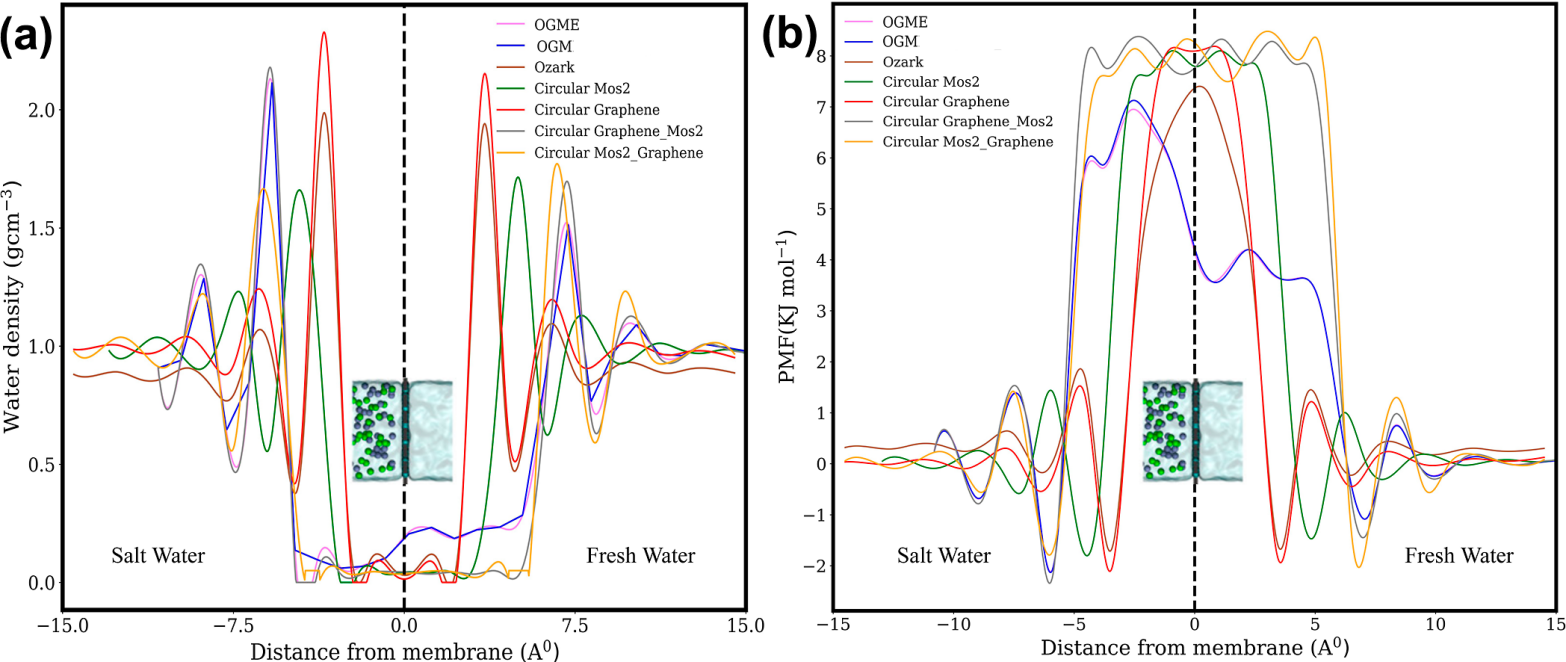
(a) Density profile of interfacial water near graphene–MoS_2_ nanopores. The location of the graphene nanopore was indicated by a dashed line. The density peak on the salt water side was magnified to highlight the observed difference. (b) Potential of mean force (PMF) for water molecules near the graphene–MoS_2_ nanopore. The OGM nanopore and OGME nanopore have lower peaks for both interfacial water density and PMF.

In order to understand the reasons behind the enhanced water flux of bilayer OGM compared with other 2D membranes, we analyzed the water structure and dynamics at both membrane surfaces and within the nanopores. Our initial focus was on examining the water density packing and the energy barrier for water transport and ions in the proximity of the membrane surfaces on both the salt and fresh water sides. It is worth noting that OGM exhibited a lower water density peak near its surface compared to other materials on both sides of the membrane (Figure 3a). Additionally, both OGME and OGM demonstrated a higher water density within the nanopore. It is important to highlight that the peak density on the salt water side was higher than that on the fresh water side, which is attributed to the applied pressure and the thickness of the two layers and heterostructure area. The lower density peaks of water near the ozark graphene nanopore indicate reduced hydrodynamic resistance at the entrance and exit of the ultrathin nanopore, facilitating enhanced water permeation. This characteristic is not limited to ozark graphene and can apply to other 2D materials, emphasizing its significance in promoting efficient water permeation.^49,50^

The PMF is used to describe the free-energy profile of water molecule transport through the graphene nanopore. In this study, we employed the Boltzmann sampling method to calculate the PMF of water molecules, represented by the equation
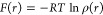
1where *F*(*r*) and ρ(*r*) denote the PMF and water density at distance *r* from the graphene–MoS_2_ nanopore in the *z* direction, respectively. *R* represents the gas constant and *T* represents the temperature. The lower peak PMF observed in the OGME nanopore (Figure 3b) depicts a reduced energy barrier for water molecule permeation. The sparser water packing near the OGM nanopore, along with its lower peak PMF for water molecules, contributes to its higher water flux. The peak density on the salt water side was higher compared to the fresh water side due to applied pressure and the thickness of the two layers and the heterostructure area. These findings suggest that the unique characteristics of the OGM and engineered nanopore, including its lower energy barrier and less dense water packing, play a significant role in facilitating the enhanced water flux observed in this study.

### Solution Density Distribution

**Figure 4:**
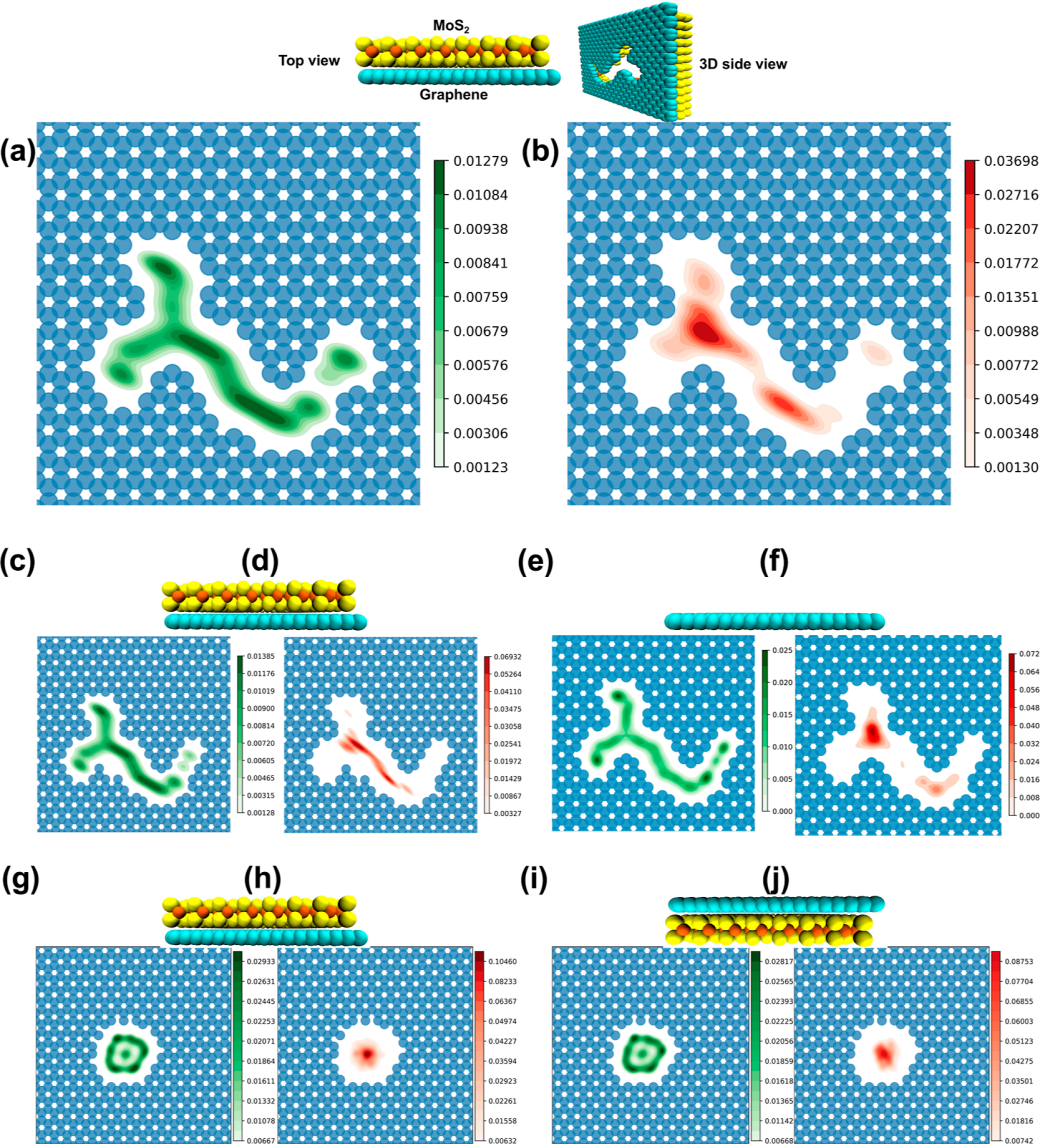
KDE plots indicate probability distribution of water and ions inside the 5 different nanoporous membranes; the green-colored area represents water and the red-colored area represents ions, inside nanopores OGME (a,b), OGM (c,d), ozark^35^ (e,f), graphene–MoS_2_ (g,h), and MoS_2_–graphene (i,j). The intensity of the color reflects the higher concentration of water or ions in a particular region. Darker colors indicate a more densely packed arrangement of water or ions in that specific area.

Analysis of water and ion distribution and dynamics within the nanopore provides insights into the exceptional water flux and ion rejection of the ozark graphene nanopore. KDE plots based on the location of molecules serve to visualize the in-pore distribution of water and ions (Figure 4). Higher color intensity indicates higher density of water or ions. Regions with higher ion density exhibit relatively lower water density. The OGM nanopore enables unobstructed water molecule transport (Figure 4a) while limiting ion transport to two specific regions within the pore (Figure 4b). By comparing the values of the OGM (Figure 4c,d) and OGME (Figure 4a,b), we have found that the OGME has higher water density at the right tip of the nanopore. Such increased water density improves the water molecule flux in the region of the engineered pore without compromising the ion rejection rate. Comparing OGM and OGME, we observe that the latter exhibits an improved distribution of packed water and increased volume of water molecules in the engineered pore region. However, in terms of ion translocation, we notice that both areas show a similar level of ions being transported from the pore on that specific region. Figure 4e–j illustrates the variations in the in-pore distribution between bilayer circular graphene–MoS_2_ and monolayer graphene and MoS_2_ (see Supporting Information Figure S3), providing a clearer understanding of their differences. The ozark nanopore’s ion-free zones, which enable the unimpeded passage of water molecules without ions, play a crucial role in maintaining a high ion rejection rate while facilitating rapid water permeation.

### Hydraulic Diameter and Porosity

**Figure 5:**
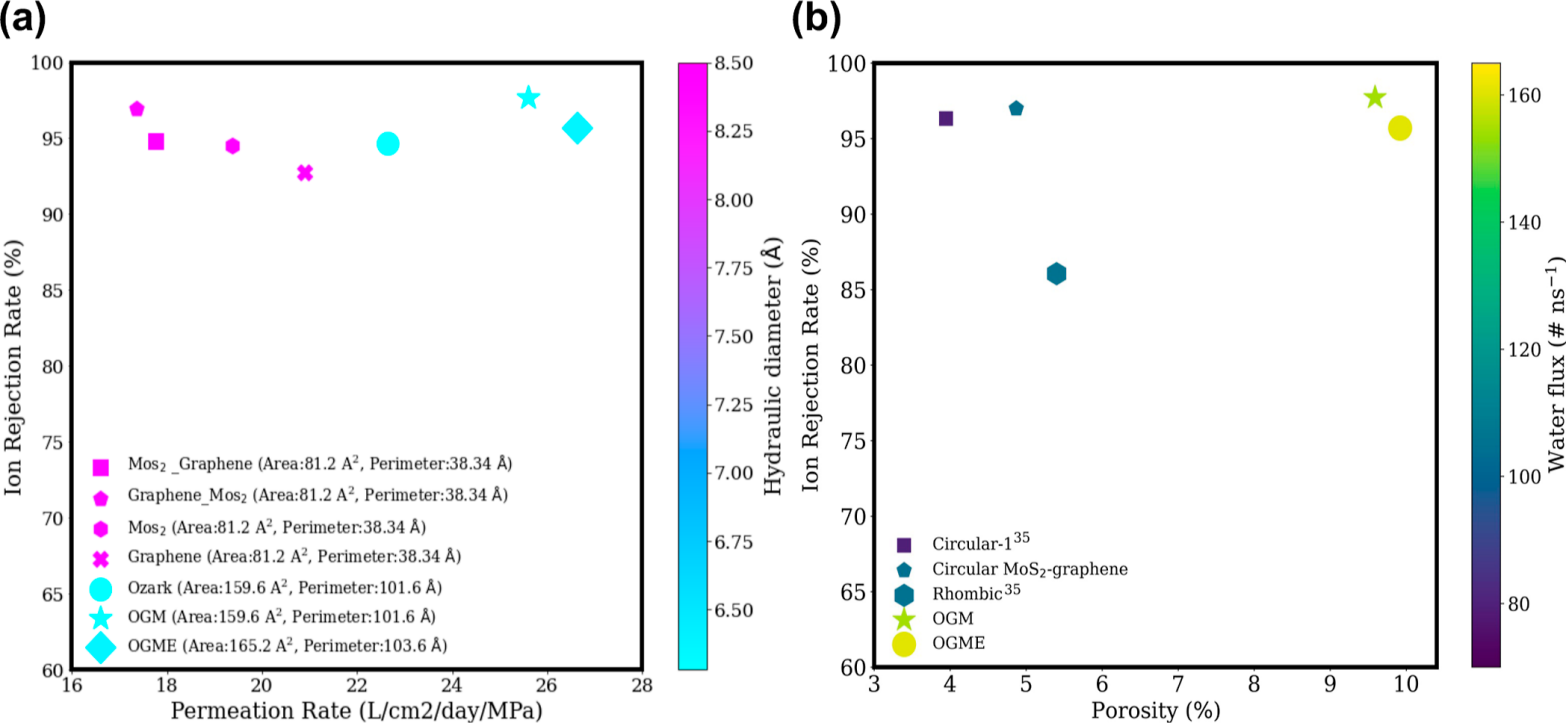
(a) Effect of hydraulic diameter on water desalination performance of nanopores. Each data point’s size corresponds to the area of the respective nanopore. The ion rejection rate is determined under a pressure of 100 MPa. The impact of porosity on the water flux and selectivity of the monolayer and bilayer of graphene–MoS_2_. (b) The plot indicates how varying levels of porosity influence the rate of water flow and the material’s ability to selectively filter out certain substances.

To quantitatively assess the impact of monolayer and bilayer nanopores on water desalination performance in terms of heterostructure and geometry, we measured the hydraulic diameter. , where *A* is the area and *P* is the perimeter of the nanopore. The observed pattern in membrane functionality suggests that hydraulic diameter can serve as a suitable criterion for modeling the behavior of noncircular and irregular nanopores.^31^ We can visualize the correlation between the hydraulic diameter and the performance of desalination (Figure 5a). The relationship between the desalination capacity of the membrane and the hydraulic diameter is nonlinear due to various factors influencing the process. Even small changes in the position of blocking atoms in the pore area, such as their elimination or addition, can result in limited fluctuations in the membrane’s desalination capacity. Therefore, the selectivity of the pore plays a crucial role in determining the overall performance.

The permeation rate is higher in nanopores with larger pore areas. For example, the OGME with the largest pore area exhibits the highest permeation rate. The OGM pore also shows a higher permeation rate compared to the monolayer ozark pore, while the ion rejection rate increases. This highlights the influence of energy barriers and water density around the bilayer pore area. We found that the key to the increasing ion rejection rate in large graphene nanopores is to reduce the hydraulic diameter. The hydraulic diameter rankings for the four nanopore areas (graphene–MoS_2_, MoS_2_–graphene, MoS_2_, and graphene) are the same (8.46 Å) > OGME (6.37 Å) > bilayer OGM and monolayer ozark (6.28 Å). Despite having a larger area compared to the circular graphene pore, the OGM has a smaller hydraulic diameter which enables it to reject more ions than the graphene pore. Additionally, even though the OGM nanopore has an approximately 95% larger area than the other nanopores mentioned (smallest hydraulic diameter), it maintains a higher ion rejection rate and water flux due to its smaller hydraulic diameter. Ions, surrounded by a hydration shell, are too large to pass through small cavities like those in the OGME (Supporting Information Figure S4). However, smaller molecules such as water can freely pass through these small cavities, and the energy barriers caused by the second layer (MoS_2_) and small cavities further reject more ions with a larger atomic radius. Therefore, reducing the hydraulic diameter by creating small cavities can enhance the ion rejection rate of large OGM nanopores. The different compositions of C, Mo, and S atoms at the edge of the pore with different vdW radii disturb the hydration shell of ions differently compared to single-layer graphene or MoS_2_. For example, Mo has a hydrophilic nature and attracts more water inside the pore so more packing of water can occur inside; however, at the entrance is carbon, which has a hydrophobic property and repels water. When ions approach the heterostructure, the graphene’s carbon at the entrance reduces the hydration shell prohibiting ions to move inside, while Mo atoms attract water, so the ions will be pushed away from entering the pore because of dehydration. In OGM, the graphene surface and carbon edges create a hydrophobic slippery surface which reduces the energy barrier at the entrance of the nanopores. After the entrance, the hydrophilic Mo edges attract the water molecules to the inside of the pore and the S terminal edges repel out the water molecules. This slip–attract–repel mechanism helps to lower the energy barriers of OGM membranes.

In order to analyze the effects of porosity on the efficiency of the bilayer OGM nanopore membrane, the water flow rate and the salt rejection rate were plotted as depicted in Figure 5b. Porosity percentage is calculated as the proportion of pore area divided by the total membrane area. For better understanding the behavior of pore shapes and the effect of adding layers on porosity, we compared bilayer OGME with a rhombic monolayer.^35^Figure 5b shows that the porosity, ion rejection, and flux of OGME has increased by 84, 11, and 52%, respectively. Increased porosity leads to a greater availability of passageways or space for water molecules to traverse through the membrane pores, resulting in an increase in water flux. However, it depends on the geometry, selectivity, and number of layers, and the percentage of ion rejection may vary in a nonlinear manner. Maintaining a balance between the porosity and mechanical strength is vital for reliable water desalination membranes. While high porosity can compromise the strength of a membrane, incorporating a heterostructure layer of MoS_2_ atoms onto a graphene membrane enhances its Young’s modulus, ultimate strength, and fracture strain.^51^ The close proximity of the layers dampens oscillations, further bolstering the membrane’s resilience. The addition of monolayer MoS_2_ to graphene has many physical and chemical effects on desalination performance. The chemical composition of Mo and S at the edge of the pore combined with the geometry of overlapping graphene and MoS_2_ potentially prohibits fouling in the membrane. In addition, MoS_2_ is intrinsically more robust than graphene and can serve as a protective layer, reducing the likelihood of membrane deformation or damage during operation. This mechanical reinforcement contributes to the long-term durability and reliability of the heterostructure membrane in practical desalination applications.^51^

## Conclusions

In conclusion, we investigate the water desalination performance of the bilayer and monolayer membranes and compare different heterostructure geometries. We successfully designed and tested a heterostructure membrane of the graphene–MoS_2_ nanopore that has a 13.1% higher water permeation rate compared to a single-layer graphene.

The enhancement of water permeation in heterostructure membranes can be attributed to the atomistic properties of both graphene and MoS_2_ that decrease the energy barrier for water molecule passage. KDE plot analysis of ion distribution within the bilayer nanopore reveals the presence of ion-free zones, where water molecules can pass through while ions are rejected. This unique feature explains the nanopore’s high ion rejection rate, which is specific to the heterostructure membrane. Furthermore, our study demonstrates that nanopores with a small hydraulic diameter in heterostructure membranes can maintain high ion rejection and permeation rates despite having a large pore area. Overall, the bilayer graphene–MoS_2_ heterostructure exhibits superior water desalination performance in terms of the water permeation rate and ion rejection, making it a promising option for significantly improving the energy efficiency of RO water desalination processes.

## Supporting Information Available

LJ potentials of atoms in MD simulation, calculations of pore area and perimeter of nanopores, bilayer and monolayer comparison in terms of ion rejection fluctuations vs pressure, KDE of monolayer graphene and MoS_2_, and effect of the hydration shell on the engineered pore and ion rejection (PDF)
