# Supplementary material for: Fast Water Desalination with a Graphene–MoS2 Nanoporous Heterostructure
Source: ACS Appl Mater Interfaces. 2024 May 20;16(22):29355–63. doi: 10.1021/acsami.4c01960 (PMC11163393; doi:10.1021/acsami.4c01960)
Supplement: Supplementary file 1 — am4c01960_si_001.pdf [file am4c01960_si_001.pdf]

# Fast Water Desalination with Graphene-$MoS_2$ Nanoporous Heterostructure: Supplementary Information

Omid Barati Farimani, Zhonglin Cao, and Amir Barati Farimani*

*Department of Mechanical Engineering, Carnegie Mellon University, Pittsburgh, USA 15213*

E-mail: barati@cmu.edu

## Contents

**1 Lennard-Jones potentials for MD simulation** **S2**

**2 Pore area and perimeter of nanopores** **S3**

**3 Bilayer and monolayer comparison in terms of ion rejection fluctuations vs pressure** **S4**

**4 KDE Plot of monolayer graphene and $MoS_2$** **S5**

**5 Effect of hydration shell on engineered pore and ion rejection** **S6**

**6 Effect of saline water ion types on OGM nanopore.** **S7**

References **S8**

# 1 Lennard-Jones potentials for MD simulation

The tabulated LJ potentials (12-6) were utilized in MD simulations to generate the dataset. The force field between different atom types was determined using the Lorentz-Berthelot rule.[1–3]

Table S1: 12-6 Lennard-Jones potentials

| Interaction | $\sigma$ (Å) | $\epsilon$ (kcal mol$^{-1}$) |
|---|---|---|
| C-C[1] | 3.3900 | 0.0692 |
| Mo-Mo[2] | 4.2000 | 0.0135 |
| S-S[2] | 3.1300 | 0.4612 |
| O-O[1] | 3.1656 | 0.1554 |
| H-H[1] | 0.0000 | 0.0000 |
| K-K[3,4] | 2.8384 | 0.4297 |
| Na-Na[3,4] | 2.1600 | 0.3526 |
| Cl-Cl[3,4] | 4.8305 | 0.0128 |

# 2 Pore area and perimeter of nanopores

The areas of graphene nanopores are determined using the computer vision method implemented with the OpenCV package.[5] For each nanoporous graphene membrane, a scatter plot is created by considering all atoms within the range of 0Å $\leq x \leq$ 40Å and 0Å $\leq y \leq$ 40Å. The diameter of each atom corresponds to the $\sigma$ value of carbon (3.39Å as stated in Table S1). The graphene membrane image has a pixel dimension of 360 × 360. The openCV package identifies the pore area as a contour (Figure S1), and the area and perimeter of the pore are calculated in pixels. The pore area is then converted to $Å^2$ using the ratio: 1 pixel = $(40/360)^2$ $Å^2$, while the perimeter is converted using the ratio: 1 pixel = (40/360) Å. The results including the perimeter, area, and hydraulic diameter of each Graphene-$MoS_2$ nanopore, are presented in Table S2.

To assess atom selectivity, modifications were made by removing/adding atoms around the pore, aiming to enhance water permeation and ion rejection (Engineered). The optimal locations for these modifications were determined based on the atomic hydrogen shell (Figure S2b and Figure S4).

Note: Table S2 column 4, provides tolerance values calculated by the OpenCV package, accounting for the arrangement and size of atoms in graphene and MoS2 surrounding the pore, with an approximate range of ±1.

Table S2: For each graphene-nanopore, calculations were performed to determine the perimeter, area, and hydraulic diameter.

| | OGME pore area | OGM pore area | Graphene - $MoS_2$, $MoS_2$ - graphene, $MoS_2$, graphene pore area |
|---|---|---|---|
| Perimeter (Å) | 103.6 | 101.6 | 38.35 ±1 |
| Area ($Å^2$) | 165.14 | 159.57 | 81.15 ±1 |
| Hydraulic diameter (Å) | 6.37 | 6.28 | 8.46 ±1 |

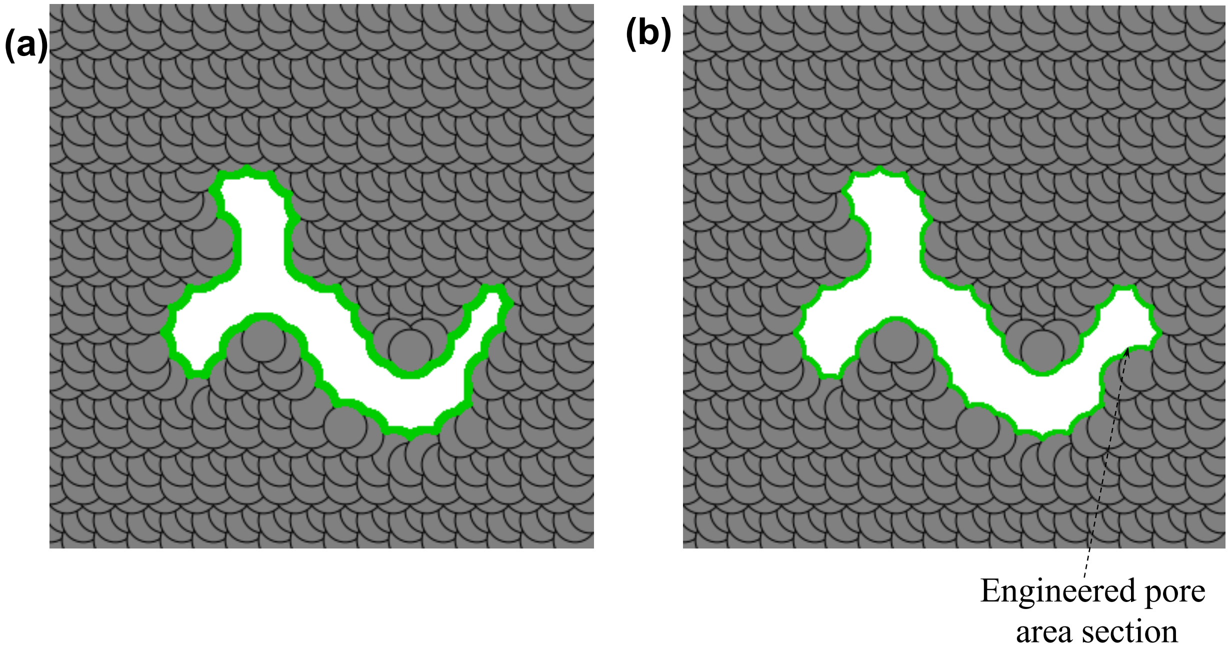


Figure S1: A scatter plot was generated to visualize the distribution of carbon atoms within the nanoporous graphene membrane. The diameter of each atom corresponds to the $\sigma$ value of the Lennard-Jones potential for carbon.**(a)** OGM pore area encircled by green contour is detected with openCV package. **(b)** OGME encircled by green contour is detected with OpenCV package depicted removing atoms of specific region in membrane.

## 3 Bilayer and monolayer comparison in terms of ion rejection fluctuations vs pressure

To gain better insights into the performance of different simulations, a comparison was made between importance of monolayer-monolayer, bilayer-bilayer, and bilayer-monolayer nanopores. This comparison revealed the impact of heterostructure bilayer nanopores on ion rejection, showing whether it improved or worsened with increasing pressure (values above zero indicate better rejection rates, while values below zero indicate worse rates). For instance, when comparing OGM with monolayer ozark at pressures of 100, 150, and 200 MPa, the ion rejection rate increased by 3.1%, 7.9%, and 13.4% respectively. These findings demonstrate that the heterostructure ozark bilayer exhibited enhanced ion rejection and improved compatibility under increased pressure (Figure S2).

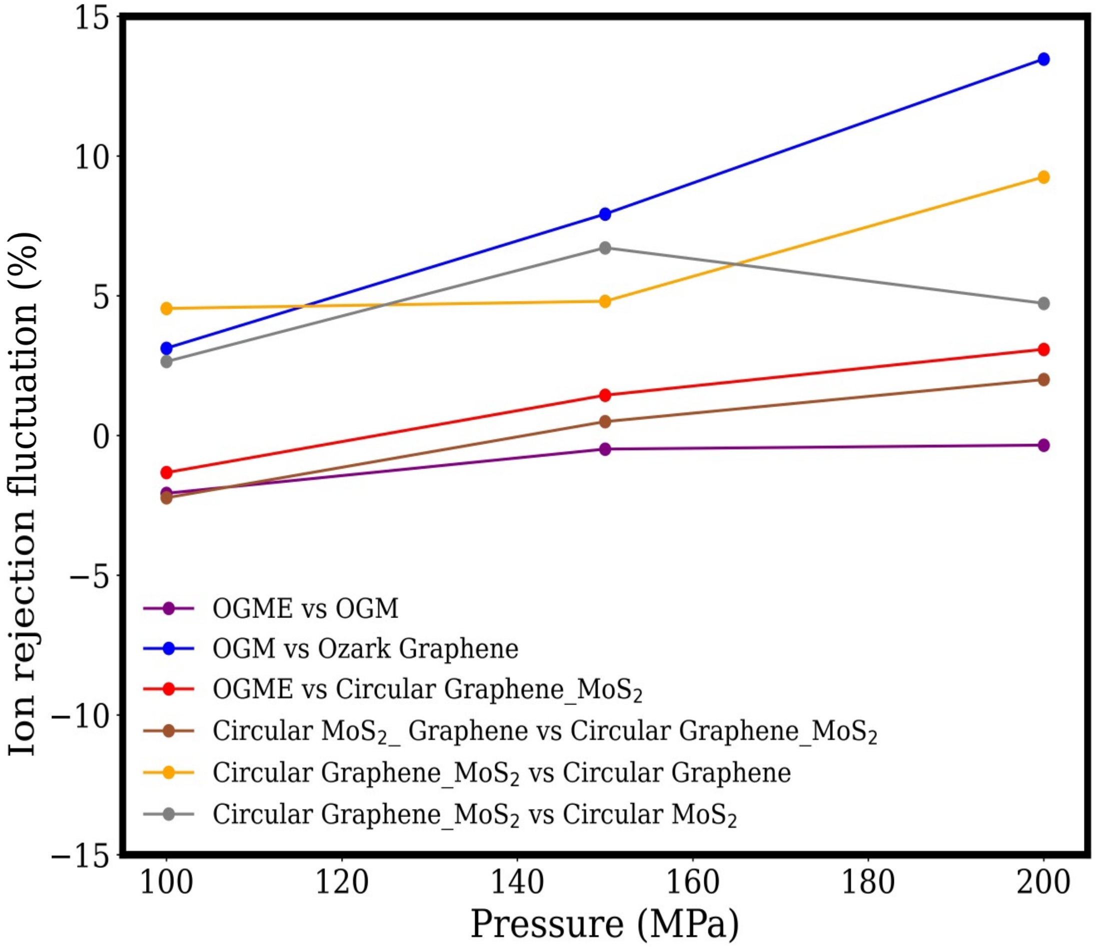


Figure S2: **(a)** Effect of bilayer and monolayer comparison in ion rejection.

# 4 KDE Plot of monolayer graphene and $MoS_2$

The KDE plot shows the probability distribution of water and ions in circular monolayer nanopores. The Green area represents water, and the Red area represents ions within the nanopore (Figure S3). Both graphene and $MoS_2$ exhibit similar sparsity of water molecules (Figure S3 a, and c), resulting in similar water flux plots (Figure 2b). However, ions are mainly concentrated in the middle of the pore (Figure S3 b and d).

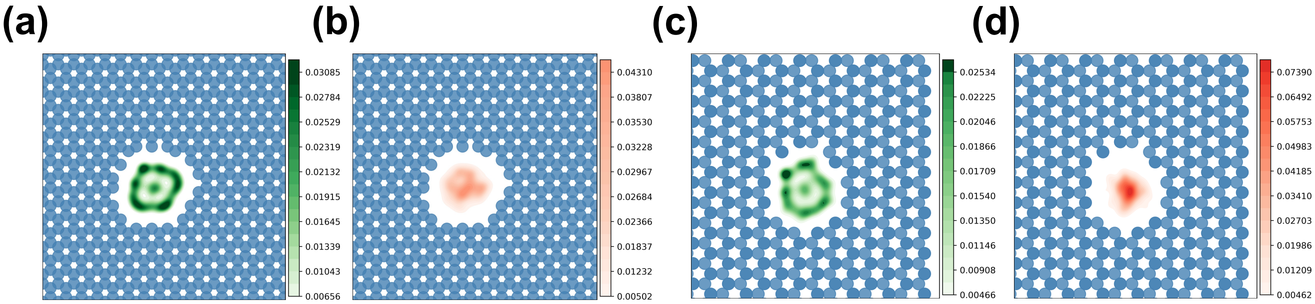


Figure S3: KDE plot graphene (**a** - **b**), and $MoS_2$(**c** - **d**)

# 5 Effect of hydration shell on engineered pore and ion rejection

Water and other polar molecules are attracted to ions, as shown in (Figure S4). The electrostatic attraction between an ion and a molecule with a dipole is called an ion - dipole attraction. These attractions play an important role in the dissolution of ionic compounds in water and can not translocate through small cavities in OGM nanopores.[4]

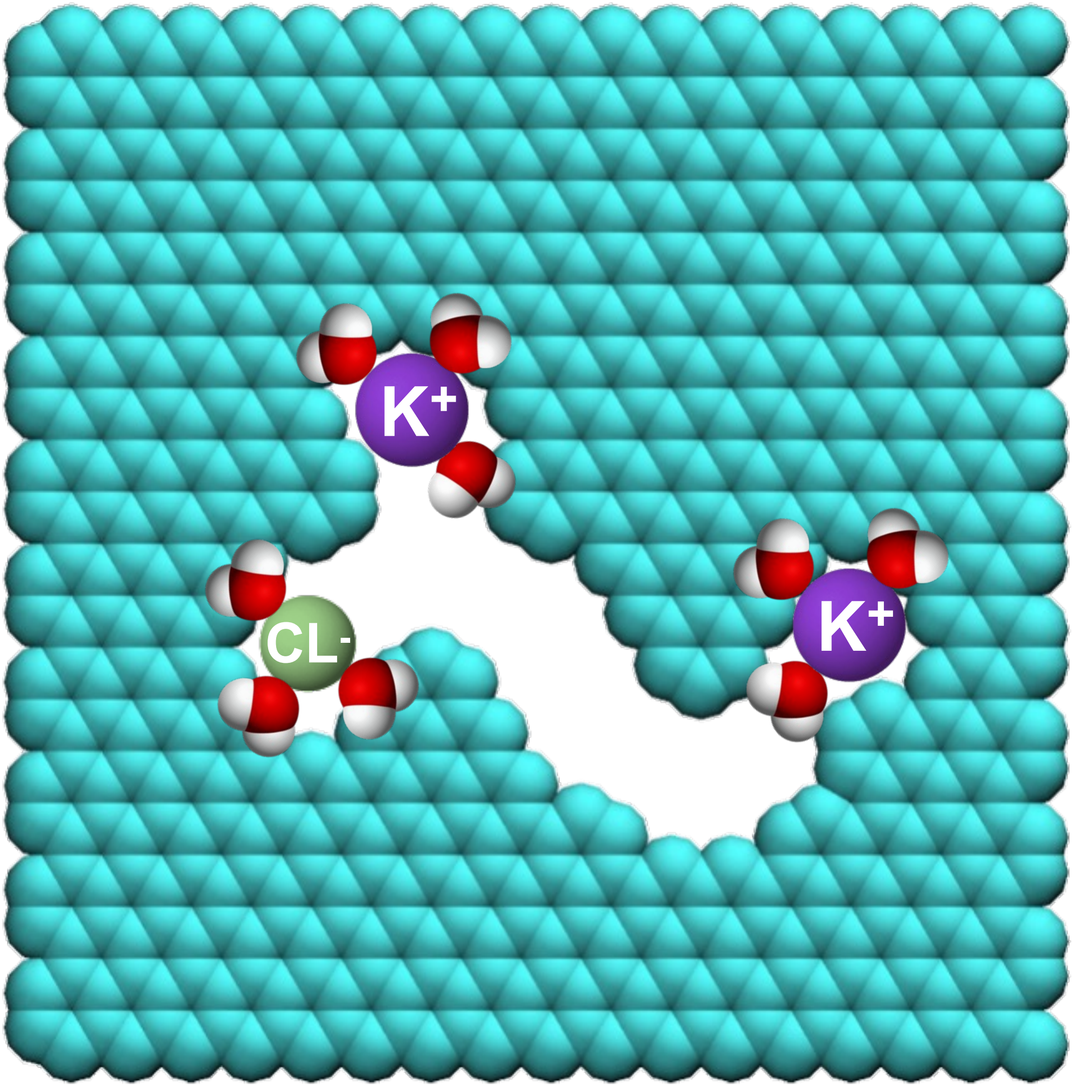


Figure S4: The cartoon illustrates ions with hydration shells being obstructed by small cavities in the engineered pore of OGM. Hydration shells, formed by water molecules (oxygen in Red, hydrogen in white), surround the ions. The ions, along with their hydration shells, are too large to pass through the small cavities.

# 6 Effect of saline water ion types on OGM nanopore.

NaCl like KCl is another common salt in seawater. We run 4 simulations at each pressure ranging from 100 to 200 MPa with 2.28 M of NaCl as the saline water and the OGM nanopore. The water flux and ion rejection rate is shown in (Figure S5).

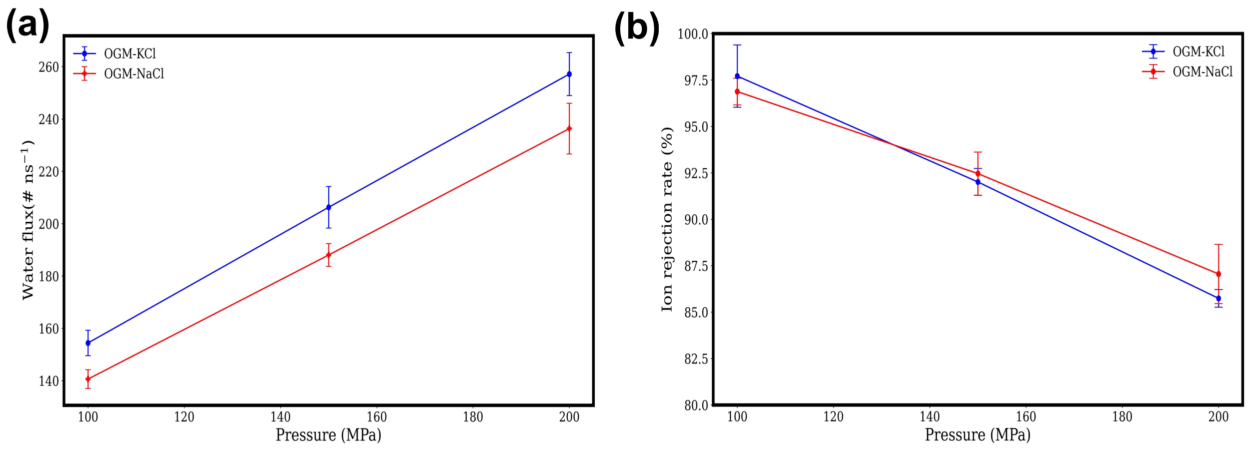


Figure S5: **(a)** Comparison of water flux for OGM nanopore between two types of salt: KCl and NaCl. **(b)** Comparison of ion rejection for OGM nanopore between two types of salt: KCl and NaCl.

# References


(1) Barati Farimani, A.; Aluru, N. R. Spatial Diffusion of Water in Carbon Nanotubes: from Fickian to Ballistic Motion. *J. Phys. Chem. B* **2011**, *115*, 12145–12149.

(2) Cao, Z.; Liu, V.; Barati Farimani, A. Why Is Single-Layer $MoS_2$ a More Energy Efficient Membrane for Water Desalination? *ACS Energy Lett.* **2020**, *5*, 2217–2222.

(3) Heiranian, M.; Farimani, A. B.; Aluru, N. R. Water Desalination with a Single-Layer $MoS_2$ Nanopore. *Nat. Commun.* **2015**, *6*, 1–6.

(4) Joung, I. S.; Cheatham III, T. E. Determination of Alkali and Halide Monovalent Ion Parameters for Use in Explicitly Solvated Biomolecular Simulations. *J. Phys. Chem. B* **2008**, *112*, 9020–9041.

(5) Bradski, G. The OpenCV Library. *Dr. Dobb's Journal of Software Tools* **2000**,
